# Case Report: Invasive and Non-invasive Hemodynamic Assessment of Coronary Artery Disease: Strengths and Weaknesses

**DOI:** 10.3389/fcvm.2022.885249

**Published:** 2022-04-25

**Authors:** Ganesh Gajanan, Saurabhi Samant, Chad Hovseth, Yiannis S. Chatzizisis

**Affiliations:** Cardiovascular Division, University of Nebraska Medical Center, Omaha, NE, United States

**Keywords:** fractional flow reserve, vFFR, CT FFR, CCTA, coronary physiology, coronary artery disease, coronary CT angiography

## Abstract

Coronary angiography has been the gold standard for assessment of coronary artery disease (CAD) and guidance for percutaneous coronary interventions (PCI). Physiology–guided PCI has shown increased safety and efficacy, improved resource utilization, and better clinical outcomes in patients with stable angina and acute coronary syndromes. The three cases presented and discussed in this report illustrate the strengths and weaknesses of the available invasive and non-invasive methods for the physiological assessment of CAD. As technology evolves, invasive non-wire-based (angiography-derived FFR) and non-invasive (FFR_CT_) modalities for the hemodynamic assessment of CAD appear to provide reliable and user-friendly alternatives to the gold standard invasive wire-based techniques. Interventional cardiologists and cardiovascular healthcare providers should be familiar with the strengths and weaknesses of the available hemodynamic assessment modalities.

## Introduction

Coronary angiography has been the gold standard for assessment of coronary artery disease (CAD) and guidance for percutaneous coronary interventions (PCI). However, coronary angiography is limited by its inability to provide information regarding the physiological significance of a given stenosis. The FAME (Fractional Flow Reserve vs. Angiography for Multivessel Evaluation) and FAME 2 trials showed that routine measurement of fractional flow reserve (FFR) in patients with multivessel CAD undergoing PCI significantly reduced the mortality and incidence of myocardial infarction at 2 years compared with standard angiography-guided PCI (1, 2). Furthermore, physiology–guided PCI has shown increased safety and efficacy, improved resource utilization, and better clinical outcomes in patients with stable angina and acute coronary syndromes (3). Currently the most common methods to assess the functional severity of a coronary stenosis are either invasive techniques, including wire-based techniques [i.e., fractional flow reserve (FFR), instantaneous wave-free ratio (iFR, Philips, The Netherlands), diastolic hyperemia-free ratio (DFR, Boston Scientific Inc., Marlborough, Massachusets, USA), resting full-cycle ratio (RFR, Abbott, Chicago, Illinois, USA)], and non-wire-based techniques [angiography-derived FFR (vFFR, Pie Medical Imaging, The Netherlands; QFR from QAngio XA, Medis, The Netherlands; FFR_angio_, CathWorks, Isarel)] or non-invasive techniques (FFR-computed tomography; FFR_CT_, HeartFlow, Redwood City, CA, USA).

The three cases presented and discussed in this report illustrate the strengths and weaknesses of the above-mentioned invasive and non-invasive methods for the hemodynamic assessment of CAD. The cutoff value for a hemodynamically significant coronary stenosis is ≤ 0.80 for wire-based FFR, angiography-derived FFR and FFR_CT_ and ≤ 0.89 for iFR.

## Case Series

### Patient 1: Discrepancy Between Non-invasive and Invasive Hemodynamic Assessment Modalities in Stented and Calcified Coronary Arteries

**Figure 1:**
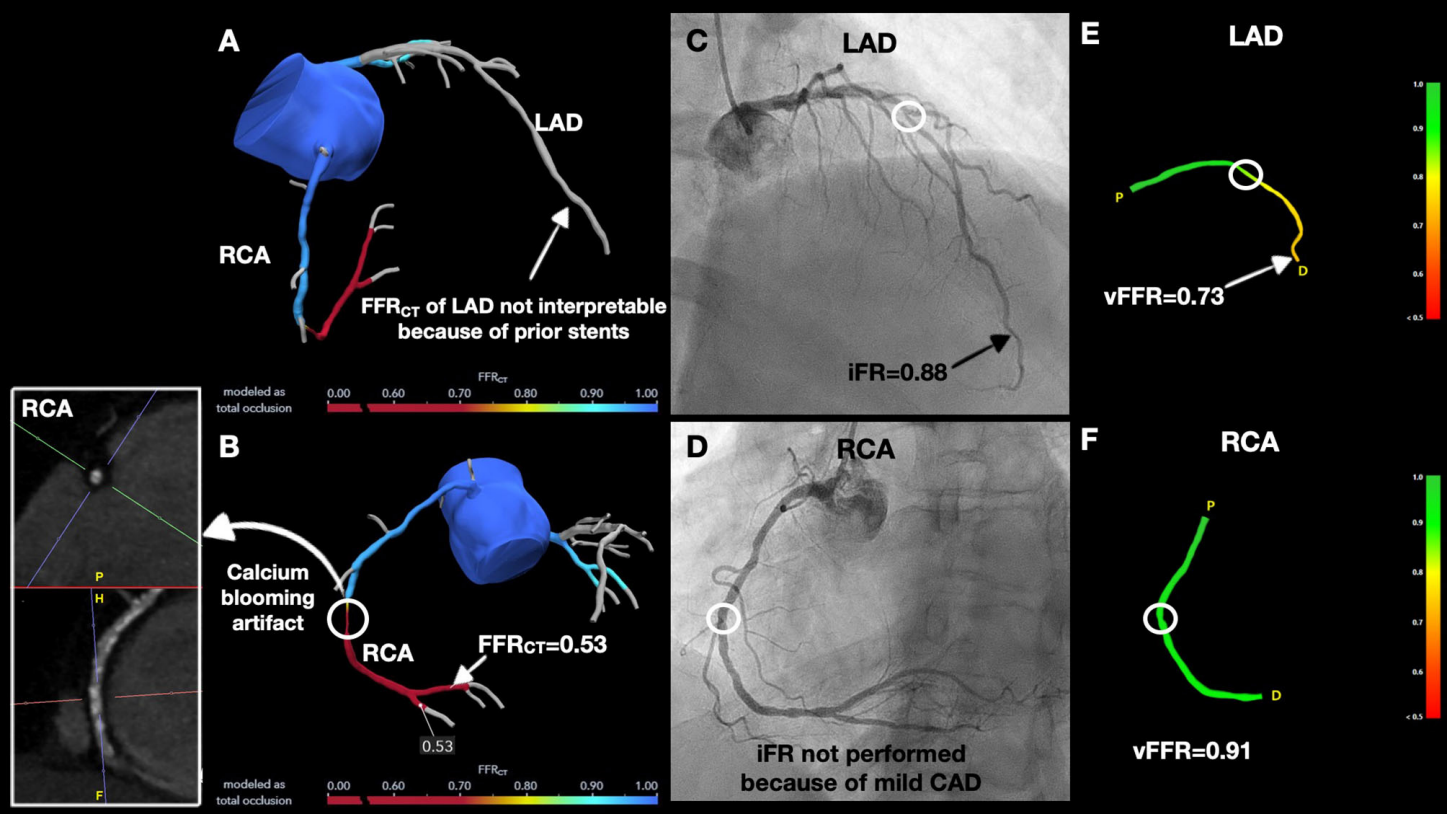
Discrepancies between non-invasive and invasive hemodynamic assessment modalities in stented and calcified coronary arteries. **(A)** FFR_CT_ of left anterior descending (LAD) could not to be interpreted because of prior stents. **(B)** CCTA showed calcium blooming artifact in right coronary artery (RCA) lesion and FFR_CT_ was significant at 0.53. **(C)** Coronary angiogram demonstrated an anatomically obstructive > 70% stenosis in the mid LAD (white circle) with instantaneous wave-free ratio (iFR) of 0.88 and, **(D)** Non-obstructive coronary artery disease (CAD) in the RCA. **(E)** Vessel fractional flow reserve (vFFR) was hemodynamically significant in distal LAD (black arrow). **(F)** vFFR of the RCA was hemodynamically insignificant.

A 64-year-old female presented with atypical chest pain for 3 months. Her medical history was notable for PCI of the proximal left anterior descending coronary artery (LAD) with two 3.0 × 12 mm drug-eluting stents (DES). She had non-obstructive CAD of the right coronary artery (RCA) and left circumflex (LCX) artery. She had a recent exercise stress echocardiogram which was negative for inducible ischemia. However, given her prior history of atypical chest pain correlating with markedly abnormal coronary angiograms, a decision was made to anatomically assess her coronary arteries with coronary CT coronary angiography (CCTA). CCTA demonstrated patent LAD stents and a focal mixed plaque was noted just distal to the stents resulting in moderate stenosis (50–70%). The proximal LCX demonstrated a mixed plaque resulting in mild stenosis (25–50%), and the mid RCA showed a long, mixed plaque, resulting in moderate stenosis (50–70%). We assessed the hemodynamic significance of the CAD with FFR_CT_ analysis. The FFR_CT_ in the LAD was not interpretable due to prior stents (Figure 1A), whereas the FFR_CT_ of the LCX was 0.90. The RCA stenosis was hemodynamically significant with FFR_CT_ at 0.53 (Figure 1B). Accordingly, we proceeded with invasive coronary angiography, which showed obstructive CAD (>70% stenosis) in the mid LAD, distal to the prior stents (Figure 1C), which was hemodynamically significant by iFR (0.88). The mid RCA showed mild diffuse disease with <50% stenosis and no focal lesions (Figure 1D). Vessel fractional flow reserve (vFFR) of the LAD was significant at 0.73 which correlated well with the invasive iFR values (Figure 1E), whereas vFFR of the RCA was 0.91 (Figure 1F). This case demonstrates the following three points: (i) Excellent correlation between invasive wire-based (iFR) and non-wire-based (vFFR) studies, (ii) Superior performance of invasive functional studies in coronary arteries with stents where FFR_CT_ is uninterpretable, and (iii) Overestimation of the hemodynamic significance of calcified plaques with FFR_CT_ (Figure 1B).

### Patient 2: Discrepancy Between Non-invasive and Invasive Hemodynamic Assessment Modalities in Severely Calcified Coronary Arteries

**Figure 2:**
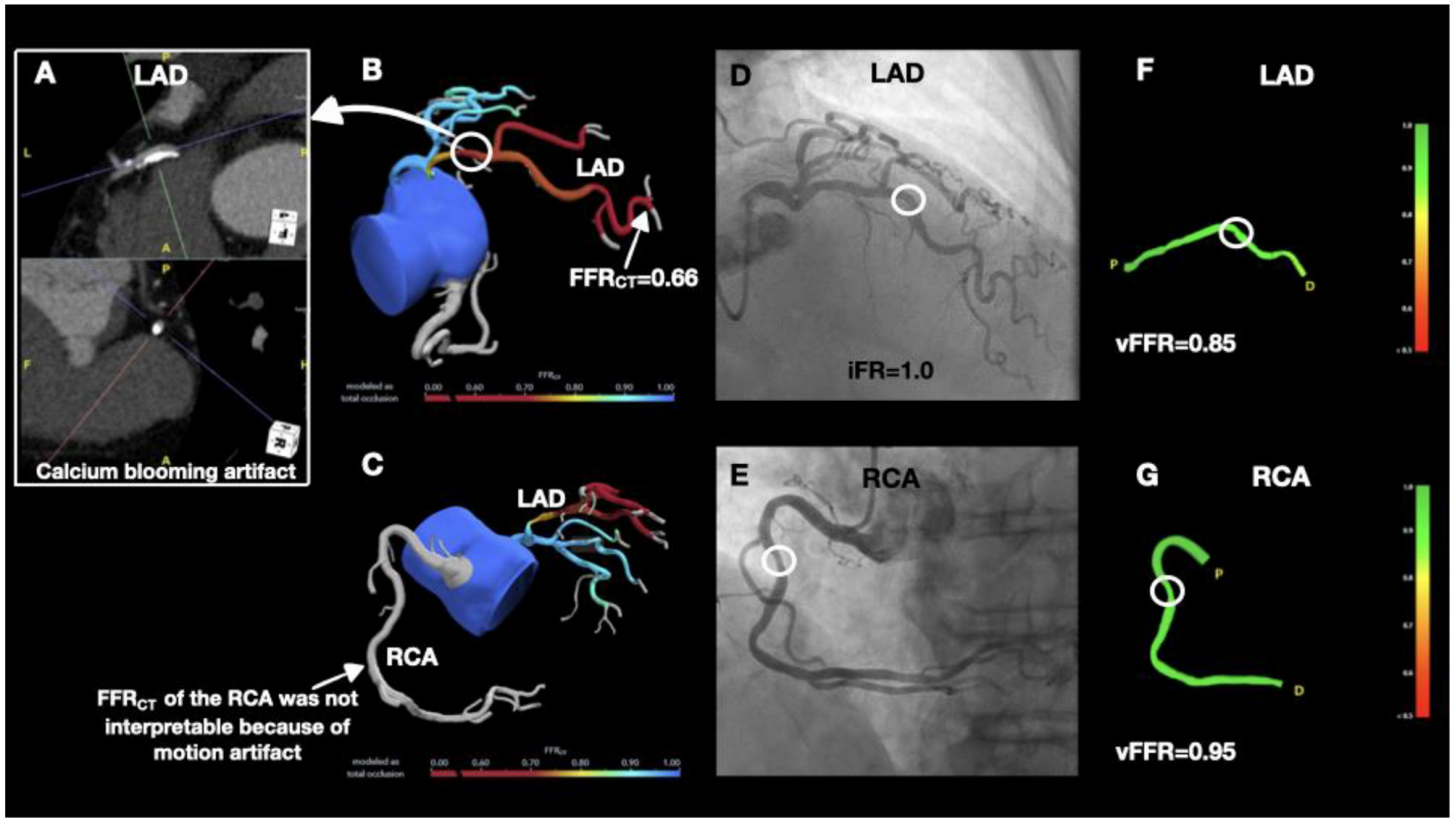
Discrepancy between non-invasive and invasive hemodynamic assessment modalities in severely calcified coronary arteries. **(A)** CCTA showing severely calcified plaque in the mid LAD. **(B)** FFR_CT_ of the LAD was hemodynamically significant. **(C)** FFR_CT_ could not be interpreted in the RCA because of motion artifact. **(D)** Coronary angiogram demonstrated <50% stenosis in the mid LAD (white circle) and iFR of 1.0. **(E)** Coronary angiography showed <50% stenosis in the mid RCA (white circle). Both, vFFR of the LAD **(F)** and vFFR of the RCA **(G)** were not hemodynamically significant.

A 76-year-old male with no prior cardiac history presented with dyspnea on exertion. Exercise stress echocardiogram was suboptimal. CCTA showed a severely calcified plaque in the mid LAD which could not be accurately quantified because of the calcium blooming artifact (Figure 2A). The total coronary artery calcium score was elevated at 1,212. A focal mixed plaque was noted in the mid RCA resulting in moderate stenosis (50–70%). FFR_CT_ analysis of the LAD was 0.66, reflecting a hemodynamically significant stenosis (Figure 2B). FFR_CT_ of the LCX was 0.88 and FFR_CT_ was uninterpretable in the RCA because of motion artifact (Figure 2C). The patient underwent invasive coronary angiography which showed anatomically non-obstructive <50% stenosis of the mid LAD with iFR at 1.0 and vFFR at 0.85 (Figures 2D,F), and non-obstructive <50% stenosis in mid RCA with vFFR 0.95 (Figures 2E,G). This case illustrates that calcium blooming or motion artifacts on CCTA can potentially preclude the accurate assessment of the severity of coronary stenoses, resulting in erroneous or uninterpretable FFR_CT_ measurements. Instead, hemodynamic assessment of severely calcified lesions can be accurately performed by vFFR, which can serve as the least invasive alternative to the iFR gold standard.

### Patient 3: Agreement Between Non-invasive and Invasive Hemodynamic Assessment Modalities

**Figure 3:**
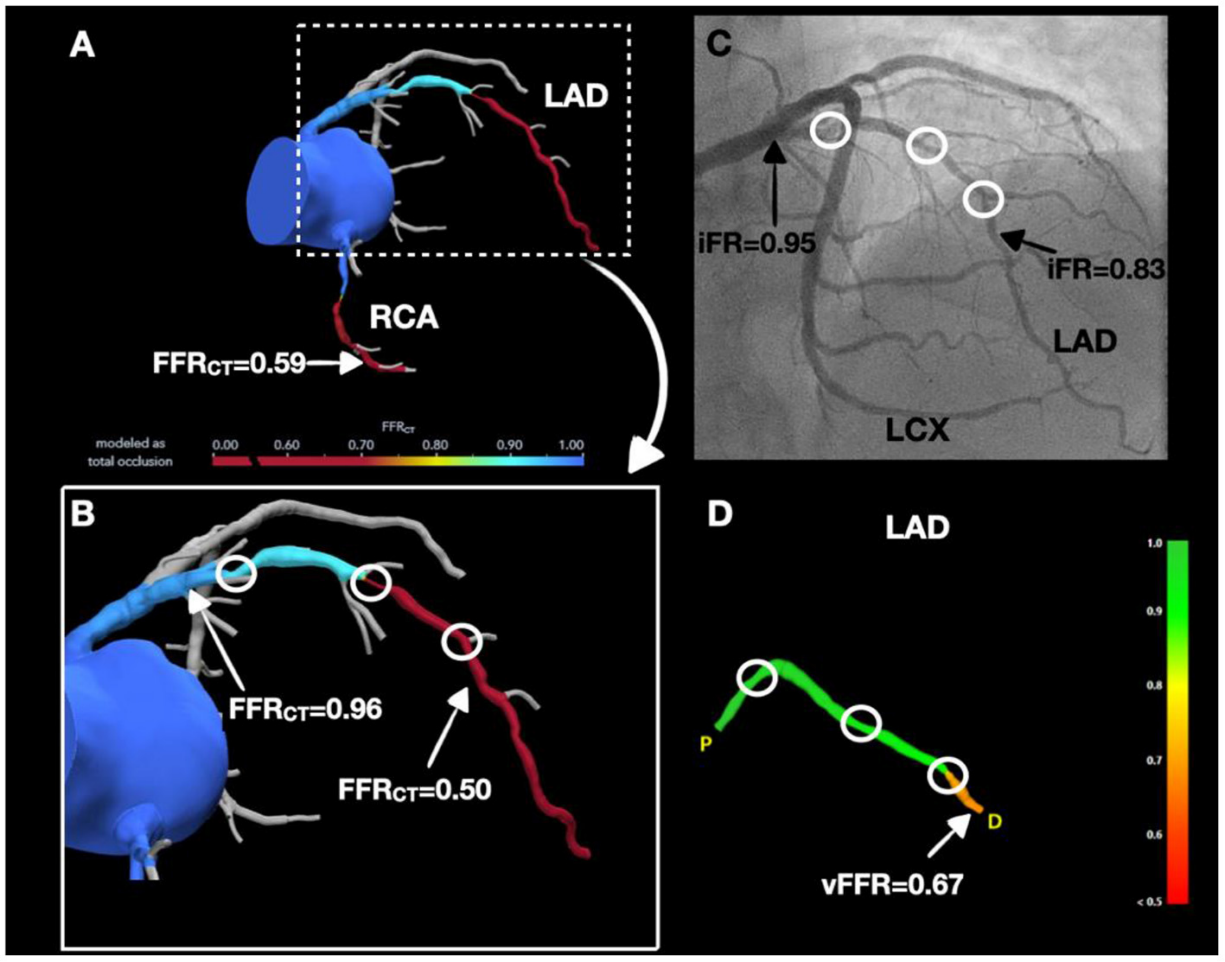
Agreement between non-invasive and invasive hemodynamic assessment modalities. **(A,B)** Significant LAD and RCA stenoses by FFR_CT_. **(C)** Coronary angiogram demonstrated 70% stenoses in the proximal and mid LAD (white circles) and iFR values of 0.95 in proximal LAD and 0.83 in the distal LAD (black arrows). **(D)** LAD stenosis was significant by vFFR (white arrow).

A 65-year-old male with a history of PCI in the left posterior descending artery (LPDA) presented with dyspnea on exertion. CCTA showed a left dominant system with a long, calcified plaque, resulting in moderate stenosis (50–70%) of the proximal LAD and mild stenosis (25–50%) of the mid LAD. A focal mixed plaque was noted in the distal LCX resulting in mild stenosis (25–50%), whereas the LPDA stent was patent. A mixed plaque was noted in a non-dominant, proximal RCA resulting in moderate stenosis (50–70%). FFR_CT_ of the LAD and RCA were hemodynamically significant at 0.50 and 0.59, respectively (Figures 3A,B). FFR_CT_ of LCX could not be interpreted because of the prior stent. Invasive coronary angiography showed 70% stenoses of the proximal and mid LAD (Figure 3C), patent LPDA stent with no evidence of obstructive disease in the LCX and a small, non-dominant RCA with proximal 70% stenosis. iFR of the LAD was 0.83 distally and during the iFR pullback jumped to 0.95 in the proximal LAD (Figure 3C). iFR pullback showed excellent correlation with FFR_CT_ values. vFFR of the LAD was 0.67, which correlated with the iFR and FFR_CT_ values (Figure 3D). This case illustrates how both non-invasive (FFR_CT_) and invasive non-wire-based techniques (vFFR) techniques correlate accurately with invasive wire-based techniques (iFR) in appropriately selected patients without severely calcified or stented coronary arteries.

## Discussion

Increasing evidence strongly suggests that presence of ischemia should help guide treatment rather than just anatomic assessment (4). Coronary angiography is limited by its ability to assess the hemodynamic significance of a given stenosis. The use of FFR to guide revascularization in patients with angina and angiographically intermediate stenoses is a Class I recommendation according to the 2021 guidelines endorsed by the major cardiovascular societies worldwide (5, 6). Despite the clinical benefits and Class I recommendation for using invasive physiology to guide coronary revascularization, its utilization in cardiac catheterization laboratories has been low due to high costs, procedural complications, contrast use and adenosine-related contraindications (Table 1) (7). Alternatively, phase-specific indices, like iFR, are equally favorable with reduced procedural times and avoidance of the adenosine-related discomfort and adverse events (8, 9). However, both hyperemic indices (FFR) and resting indices (e.g., iFR) may result in complications related to their invasive nature (e.g., coronary dissection and perforation). Non-invasive (FFR_CT_) and invasive wire-free techniques (angiographic FFR) have demonstrated comparable predictive values to FFR and iFR in determining functionally obstructive CAD with no wire-related risks, and reduced cost, contrast, and radiation exposure (10–12).

**Table 1:** Comparison between invasive and non-invasive methods of hemodynamic assessment of coronary stenoses.

	**Invasive**				**Non-invasive**
	**Wire-based**			**Wire-free**	**CCTA-based**
	**Stress indices**	**Resting indices**			
	**FFR**	**P_**d**_/P_**a**_**	**iFR, DFR, RFR**	**vFFR, QFR, FFR_**angio**_**	**FFT_**CT**_**
Cutoffs	0.80	0.92	0.89	0.80	0.80
Invasive	Yes	Yes	Yes	Yes	No
Pressure wire	Yes	Yes	Yes	No	No
Hyperemia	Yes	No	No	No	No
Contrast	Yes	Yes	Yes	Yes	Yes
Radiation	Yes	Yes	Yes	Yes	Yes
Procedural time	Increased	Decreased	Decreased	Decreased	Not applicable
Severely calcified lesions	Good performance	Good performance	Good performance	Good performance	Limited use
Stents	Good performance	Good performance	Good performance	Good performance	No
Severe motion artifacts	Good performance	Good performance	Good performance	Good performance	Limited use

*FFR, fractional flow reserve; iFR, instantaneous wave-free ratio (Philips, The Netherlands); DFR, diastolic hyperemia-free ratio (Boston Scientific Inc., Marlborough, MA, USA); RFR, resting full-cycle ratio (Abbott, Chicago, IL, USA); vFFR, vessel fractional flow reserve (Pie Medical Imaging, The Netherlands); QFR, Quantitative flow ratio (Medis, The Netherlands); FFR_angio_, Fractional flow reserve- angiography (CathWorks, Isarel); FFR_CT_, FFR-computed tomography (HeartFlow, Redwood City, CA, USA); CCTA, coronary computed tomography angiography*.

FFR_CT_ is based on the application of computational fluid dynamic analysis to a CCTA dataset under maximum computationally simulated hyperemia. FFR_CT_ is cost-effective and has lesser complication rates, thus decreasing the need for invasive coronary angiography (Table 1) (13, 14). FFR_CT_ has been noted to correlate very well with the reference standard invasive indices (i.e. FFR, iFR) (15). The biggest advantage of FFR_CT_ is that on top of the hemodynamic assessment of a given stenosis, it provides useful information on plaque morphology, and identification of high-risk plaques, which are the precursors of a significant portion of acute coronary syndromes (12). However, FFR_CT_ requires high quality CCTA images which are difficult in patients with large body habitus and suboptimal heart rates. Furthermore, FFR_CT_ might provide suboptimal results in cases with motion artifact or noise, severe coronary calcifications and prior stents.

Angiography-derived FFR software uses coronary angiography to calculate the pressure drop across the anatomical stenoses (Table 1). Multiple clinical trials including, the FAST I, FAVOR, and FAST-FFR study have validated the accuracy, reliability, and reproducibility of different angiography-derived FFR softwares, i.e., vFFR, QFR and FFR_angio_, respectively (16–19). Angiography-derived FFR has consistently shown similar accuracy to the pressure wire–based FFR in large prospective trials and cohort studies (20). Angiography-derived FFR is an easier and potentially faster method for physiology-guided assessment of coronary vessels compared to wire-based techniques (17, 21). However, angiographic FFR requires high-quality angiography in specific orthogonal views without any vessel overlap. This high-quality imaging would be difficult in severely tortuous and very small caliber vessels, obese patients, and patients with chronic kidney disease where contrast use is limited (20).

Newer non-wire based modalities are being increasingly studied. For example, myocardial blush grade reserve has been noted to closely correlate with FFR (22). As technology evolves, invasive wire-free (angiographic FFR) and non-invasive (FFR_CT_) modalities for the hemodynamic assessment of the CAD, appear to provide reliable and user-friendly alternatives to the gold standard invasive wire-based techniques. Interventional cardiologists and cardiovascular healthcare providers should be familiar with the strengths and weaknesses of the available hemodynamic assessment modalities.

## Data Availability Statement

The original contributions presented in the study are included in the article/supplementary material, further inquiries can be directed to the corresponding author/s.

## Ethics Statement

Written informed consent was obtained fromthe participants for the publication of this case report.

## Author Contributions

GG involved with the management of the patients and leading the write up of the manuscript. SS made significant contributions to writing the manuscript and proofreading. CH involved with processing of coronary CT and FFT CT images. YSC involved directly in treating the patient, mentored, and made suggestions in the preparation of the manuscript. All authors contributed to the article and approved the submitted version.

## Conflict of Interest

YSC has speaker honoraria, advisory board fees and a research grant from Boston Scientific Inc. and a research grant and advisory board fees from Medtronic Inc. The remaining authors declare that the research was conducted in the absence of any commercial or financial relationships that could be construed as a potential conflict of interest.

## Publisher's Note

All claims expressed in this article are solely those of the authors and do not necessarily represent those of their affiliated organizations, or those of the publisher, the editors and the reviewers. Any product that may be evaluated in this article, or claim that may be made by its manufacturer, is not guaranteed or endorsed by the publisher.

## Abbreviations

CAD: Coronary artery disease
CT: Computed tomography
CCTA: Computed tomography coronary angiography
DES: Drug-eluting stent
DFR: Diastolic hyperemia-free ratio
FFR: Fractional flow reserve
iFR: Instantaneous wave-free ratio
LAD: Left anterior descending artery
LCX: Left circumflex artery
LPDA: Left posterior descending artery
PCI: Percutaneous coronary interventions
RCA: Right coronary artery
RFR: resting full-cycle ratio
QFR: Quantitative flow ratio
vFFR: Vessel fractional flow reserve.
